# Supplementary material for: Are carbon emissions trading and green financial instruments synergistic? -Comprehensive quantitative research based on content analysis
Source: PLoS One. 2024 Mar 7;19(3):e0298601. doi: 10.1371/journal.pone.0298601 (PMC10919601; doi:10.1371/journal.pone.0298601)
Supplement: S1 Data — (ZIP) [file pone.0298601.s001.zip › Data/地级市Z_F工作报告.zip!/░▓╗╒/┬φ░░╔╜╩╨/└┤╫╘╣1⁄2╓┌║┼í╛┬φ┐╦╩2╛▌═°í┐_2009.docx]

# **政府工作报告（2009）**

【字体大小：[大](http://zwgk.mas.gov.cn/openness/detail/content/javascript:doZoom(18)) [中](http://zwgk.mas.gov.cn/openness/detail/content/javascript:doZoom(15)) [小](http://zwgk.mas.gov.cn/openness/detail/content/javascript:doZoom(14))】

市长 周春雨

各位代表： 


    现在，我代表市人民政府向大会作工作报告，请予审议，并请市政协委员和其他列席人员提出意见。

    一、抢抓机遇、攻坚克难、

    奋力开拓的2008年

    刚刚过去的2008年，是马鞍山改革发展进程中极不平凡、经受挑战并取得重大成就的一年。一年来，面对自然灾害和突发事件的严峻考验，面对国际金融危机对我国经济的巨大冲击，在省委、省政府和市委的坚强领导下，全市上下认真贯彻党的十七大、十七届三中全会和胡锦涛总书记两次视察安徽重要讲话精神，全面落实科学发展观，较好地完成了市十四届人民代表大会一次、二次会议确定的年度目标任务，全市经济社会发展主要指标实现“十大突破”，与长三角发展差距进一步缩小，在推进“又好又快新跨越、率先全面达小康”征程上迈出了坚实的步伐。

    预计全市实现生产总值突破600亿元，达618亿元，比上年增长16%。人均生产总值突破7000美元，达7019美元。财政收入突破100亿元，达110.2亿元，增长20.5%。全社会固定资产投资突破400亿元，达402亿元，增长21.3%。规模以上工业销售收入突破1000亿元，达1200亿元，增长33.1%。中小企业总数突破1万户，提前2年实现中小企业发展目标。社会消费品零售总额突破100亿元，达105亿元，增长21.3%。实际利用外资突破5亿美元，达5.1亿美元，增长37.4%。实际利用内资突破200亿元，达220亿元，增长47.8%。全面小康实现程度突破九成，达91.3%，比上年提升4.8个百分点。

    （一）积极应对复杂多变的宏观形势，工业经济保持快速增长。一年来，我们认真贯彻中央宏观调控政策，在抢抓机遇中努力推动经济加速增长，在克服困难中全力保持经济平稳运行。抓住上半年宏观经济环境总体宽松的有利时机，我们强化经济运行调节，确保各种生产要素供给，千方百计支持企业扩大生产和销售。上半年全市生产总值增幅位居全省第一，规模以上工业增加值保持全省第二。在下半年特别是第四季度经济困难明显加重、我市支柱产业遭遇冲击较大的情况下，我们按照中央“出手要快、出拳要重、措施要准、工作要实”的要求，及时出台促进经济增长的一系列政策措施，着力防止经济出现大的起伏，较好地保持了经济平稳较快发展的势头。

    着力实施工业强市战略，狠抓重点项目建设。积极帮助企业及时化解生产要素制约，采取有效措施推动银企对接，全年为各类企业减税、减费、让利20多亿元，促进工业经济加快发展。马钢紧紧围绕“快速转变发展方式、显著提高经营效果”工作主题，灵活调整生产经营思路，大力提升品种质量，扎实开展降本增效，在下半年钢材市场价格持续大幅下跌的困难条件下，全年销售收入仍超过700亿元。车轮扩能改造项目竣工投产，成为全球最大车轮生产基地；大型支承辊和锻钢生产线项目快速推进，“十一五”后期结构调整项目前期工作全面启动。华菱3万辆重卡扩建项目通过国家发改委核准，新产品开发和市场开拓成效明显。山鹰20万吨高强瓦楞原纸技改项目竣工，马鞍山发电厂两台60万千瓦机组扩建项目前期工作公司成功对接。加大专精特新企业培育力度，安徽冶金装备制造基地挂牌成立，机械刃模具、食品加工等产业集群逐步壮大。全年完成工业性投资245亿元，增长15.4%；新增规模以上工业企业120户，规模以上工业实现增加值375亿元，增长22%。

    加大自主创新和节能减排力度，加快工业经济结构调整步伐。马钢启动实施高速列车车轮等国家级自主创新项目，成功开发X80管线钢等高端产品。矿院国家“十一五”科技支撑计划二期重大项目获批，国家重点实验室申报工作取得重大进展。国家钢铁及制品质量监督检验中心开工建设。“863”新材料基地主体园区基本建成，2个计划引导项目通过科技部验收，茂文科技园等项目开工建设。成功举办第三届安徽（马鞍山）产学研合作项目对接会，与中科院合肥物质科学研究院建立全面合作关系。马钢、矿院各有1个项目荣获“国家科技进步二等奖”。全市有1个项目获国家火炬计划立项，210多项专利获得授权，7个产品被授予“安徽名牌”。新增高新技术企业12家、高新技术产品34个，总数分别达88家、129个；实现高新技术产业产值340亿元，增长25%。加强钢铁、电力、造纸和建筑等重点行业节能减排工作，实施58项节能减排重点工程，45项建成发挥环境效益。重点企业污染源在线监控实现全覆盖，第一次污染源普查工作顺利完成。永久性切断“五小”、“两高”企业供电，列入淘汰的落后产能设备基本拆除完毕，460万吨钢铁产能置换项目获准实施。市第二污水处理厂运营模式顺利转换，东部污水处理厂、慈湖污水处理厂前期工作加快推进。全市万元生产总值综合能耗下降4.1%，主要污染物排放量控制在省下达指标以内。

    （二）率先开展城乡一体化综合配套改革，新农村建设加快推进。立足我市农村经济社会发展现状，制定出台城乡一体化综合配套改革总体方案和8个专项方案，对今后几年工作作出具体安排。花山区改革试点工作扎实开展，张庄中心村首批农民公寓开工建设，濮塘中心村规划正式启动，霍里镇“镇改街”获得批准。农村综合改革向纵深推进。集体林权制度改革进入扫尾阶段。兽医管理体制改革全面完成。种植业政策性农业保险投保率居全省前列。农村集体土地上房屋权属登记发证工作取得积极进展。第七届村民委员会换届选举顺利完成。实施结对帮扶制、试点镇村综合考核末位淘汰制，新农村建设试点工作稳步推进。

    着力发展现代农业，农村经济加快增长。全年实现农业增加值25亿元，增长22%。粮油全面增产，粮食总产创历史新高。苗木花卉等优势农产品基地规模进一步扩大，河蟹、畜禽规模养殖比重持续上升。农业产业化快速推进，全市有2家龙头企业营销收入超10亿元，黄池食品集团成为国家级农业产业化龙头企业，新增农民专业合作社30家。在建、续建“三资”农业项目109个，实际到位资金4.2亿元。万头奶牛和优质生猪养殖被列入全国农业标准化示范区项目，全年新增绿色食品7个、无公害农产品11个。

    加快发展公共事业，农村生产生活条件持续改善。高度重视农村留守儿童教育工作，全面完成农村中小学D级危房改造任务。建成标准化乡镇卫生院5所、村卫生室52所。20户以上自然村“村村通”有线电视工程在全省率先完成。调整新型农村养老保险农民参保缴费政策，全市新农保参保率比上年提高8个百分点。农民工工资支付长效机制进一步完善。澄湖路、青黄路竣工通车，建成通乡油路40公里，农运班线覆盖率达92.5%。实施病险水库、涵闸除险改造37座，大公圩排涝泵站改造全面建成。农村生活垃圾集中处置在市郊基本实现，并在当涂县6个乡镇全面推开试点。实施“新网工程”，组建农资消费合作社，供销社开放办社积极推进。

    （三）大力促进服务业跨越发展，第三产业发展水平得到提升。制定现代物流业发展规划和扶持政策，推动生产性服务业加快发展。长江港口公共码头开港，天顺码头投入试运行，长运物流港、人头矶码头开工建设，港口年吞吐量创历史最高水平。中石化加油加气合建站布点建设快速推进，中油高佳加气子站竣工运营，出租车“加气难”问题有所缓解。成功举办中国（马鞍山）刃模具暨剪折机床博览会。省内一流的机械??期工程竣工，塘南水产品专业市场建成运营。软件产业成为新亮点，与上海徐汇国家级软件基地交流合作机制全面建立，北大软件技术人才实训基地成功创办，佳达创意软件园建设进展顺利，全市累计兴办软件企业20多家。各金融机构积极优化信贷结构，创新服务方式，在服务地方经济中实现长足发展。去年末金融机构贷款余额329亿元，比上年增加25亿元。农村合作银行荣获“全国小企业金融服务先进单位”称号，改制组建全国第一家市级农村商业银行获得批准。邮政储蓄银行马鞍山分行挂牌运营。浦发银行马鞍山支行获准筹建。徽商期货落户马鞍山。融资担保体系进一步完善。

    推动消费结构升级，生活性服务业水平明显提升。商贸流通业蓬勃发展，跃居2008年福布斯中国大陆最佳商业城市百强榜第77位。沃尔玛主体工程封顶，大华国际广场二期、花山美居项目加快推进，新都市场一期工程完工，早餐工程顺利实施。一批生态旅游项目开工建设，李白墓园成为国家3A级旅游区，蒙牛乳业（马鞍山）公司、当涂园艺村分别被命名为全国工业、农业旅游示范点。按照“鼓励首购房、小套房，搞活二手房、二套房”的总体要求，出台十二条政策措施，促进房地产市场平稳健康发展。全年新开工房屋面积211万平方米，竣工面积148万平方米。加强保障性住房建设，6000多套安置房、312套廉租房交付使用，廉租住房补贴人数、额度大幅增长。21个危旧房改造工程加快推进，15个老旧小区综合整治全部完成。

    （四）深入推进改革开放，经济发展动力与活力进一步增强。以纪念改革开放30周年为契机，继续深化重点领域改革。制定出台国有建设用地使用权净地出让制度，修订完善征地拆迁补偿安置办法。按照“能放则放、规范分配、促进发展、逐步完善”的原则，制定出台市区财政体制改革办法，从体制机制上进一步调动三区加快发展的积极性。整合公立医疗资源，组建市立医疗集团，以政事分开、管办分开为重点的医疗卫生体制改革在全省率先实施。出台深化文化体制改革若干意见，市工人剧场、人民会堂完成改制。事业单位岗位设置试点积极开展，马和轮渡转企改制基本完成。百货公司资产重组工作加快推进，三大市属国有投资公司资产实现保值增值。企业上市和再融资在资本市场低迷的情况下取得突破，泰尔重工、鼎泰稀土上市通过证监会预审，马钢认股权证二次行权募集资金30多亿元。

    坚持“引进来”和“走出去”相结合，对外开放水平进一步提升。成功举办直接对接世界500强驻中国代表处的马鞍山（北京）投资环境说明会，精心组团参加徽商大会、中博会等重大招商活动。修订完善考核办法，强化招商引资工作的针对性和有效性，利用内外资实现新突破。开发园区承接产业转移能力进一步增强。市经济技术开发区南区建设全面启动，科达机电、稳润光电等一批大项目成功引进，达利食品一期工程顺利竣工。慈湖经济开发区基础设施日趋完善，立白华东基地一期工程建成投产。对外贸易逆势上扬，完成进出口总值32亿美元，增长36.2%，机电产品和高新技术产品出口增幅均超过70%，被商务部授予“中国剪折机床出口共建基地”。边防检查站顺利建成，口岸即将对外籍轮开放，马鞍山港成为首批与台湾直航港口之一。对外承包工程和劳务合作营业额超1亿美元，十七冶境外承包工程合同额位居全省外经企业前列。积极参与泛长三角区域分工与合作，共同签署南京都市圈综合交通发展规划备忘录和交通项目对接协议。新增美国吉狮堡、墨西哥特拉内潘特拉2个国际友好城市，对外交往进一步扩大。

    （五）加速推动县区发展和全民创业，经济发展协调性持续改善。在规划、土地、财政等方面给予县区大力支持，着力激发县区加快发展的积极性、主动性和创造性。当涂县实现生产总值120亿元，增长18%；完成财政收入15.6亿元，增长38.3%；大唐当涂电厂一期、国星生化等一批重点项目建成投产；县职教中心新校区全面建成，博望三杨村被评为全省唯一“国家级生态村”。三区立足区情，发挥各自优势，发展呈现齐头并进态势，合计实现财政收入近14亿元，增幅均超过30%。花山区实现社会消费品零售总额占全市份额一半以上，商贸旅游园和工业集中区完成合并，软件园孵化器一期工程投入运营，荣获“全国科技进步考核先进区”称号。雨山区新增销售收入超亿元企业3户，双益车轮等一批项目建成投产，再次被授予“全国科普示范城区”称号。金家庄区百亿级金属加工制造产业集群初具雏形，方圆大型回转支承等项目竣工投产，区档案馆成为“国家二级档案馆”。

    着力优化发展环境，全民创业深入推进。进一步减免9个部门20项行政事业性收费，减轻企业和创业者负担。放宽企业登记限制，下放注册登记权限，在全省率先开展股权质押合同鉴证。设立全省首个中小企业服务中心和大学生创业工作站，加快建设农民创业园，创建省级创业基地12个，累计建成标准厂房40多万平方米，入驻创业实体近700个。全年新发展个体工商户9842户、私营企业1738户，个私经济纳税额超过18亿元，发展非公经济工作受到省委、省政府表彰。

    （六）进一步加大城市建设管理力度，市容市貌明显改观。扎实推进城乡规划和建设，着力提升城市承载功能。完成全市土地利用总体规划修编工作，启动城市消防、蓝线等专项规划编制。一批关系马鞍山区域定位、长远发展和整体形象的重点工程加快推进，长江公路大桥、宁宜城际铁路、芜申运河航道整治工程开工建设，马濮路改扩建工程全面启动，东环路高速化改造前期工作取得积极进展。强力实施雨山路环境综合整治工程，成为集交通、生态、休闲于一体的城市标志性景观大道。马向路市政化改造有序推进，江东大道软基路段、湖东路改造一期工程顺利实施。湖北路滨湖休闲游园动工兴建，林业生态等绿化工程成效显著，“全国绿化模范城市”正式授牌。向山天然气供气等工程加快实施，全年完成小城镇建设投资13亿元。与国家开发银行签署合作意向，城市基础设施、生态环境建设等领域41个项目获得金融贷款支持。

    城市管理工作进一步加强，环境定量考核连续多年保持全省之首。备受社会各界关注的慈湖河流域生态环境综合整治全面展开，上游化工企业、尾矿库污染治理取得阶段性成效。强力推进江河堤防安全整治，影响江滩河道行洪安全的各类违法建设基本清除，长效管理机制正在逐步落实。制定金家庄部分地区居民搬迁总体方案，江边地区居民搬迁前期工作扎实开展。雨山河等4条水系整治全面完成，城市防汛排涝调度中心建成使用。强化户外广告、门头招牌管理，规范建筑垃圾、渣土运输行为，近郊违法建设拆除力度不断加大。向山垃圾场二期工程开工建设，45座公厕免费向市民开放。

    （七）以加强和改善民生为重点，社会建设再谱新篇章。组织实施40项民生工程，当年投入资金7.6亿元，惠及全市城乡居民。开展城乡统筹就业试点，实施就业信息进村入户工程，强化城乡困难人员就业帮扶，提高再就业培训补贴标准，创业培训范围继续扩大。全年新增城镇就业3.1万人，农村劳动力转移就业1.9万人，城镇登记失业率为3.7%。出台实施城镇老年居民养老保险制度、事业单位养老保险暂行办法，实现养老保险制度城乡全覆盖。城镇居民医疗保险与新型农村合作医疗实现有效整合，城乡一体化基本医疗保障体系初步建立。失业保险市级统筹成功实施。稳步提高城乡低保标准和人均补差，取消城乡医疗救助起付线和病种限制，“五保”集中供养率达40%。开展多种形式的帮扶和送温暖活动，切实解决困难群众的生活问题。社会福利、慈善事业持续健康发展。截至去年底，我市覆盖城乡居民的社会保障体系基本建立。全年城市居民人均可支配收入18200元，农民人均纯收入6900元，分别增长12.8%和12.3%。

    “文化发展年”活动扎实开展，文明创建富有成效。市博物馆和新图书馆建成试开馆，大剧院主体工程进入扫尾阶段。成功举办’2008马鞍山中国李白诗歌节。马钢被授予“全国企业文化建设优秀单位”称号。文化信息资源共享工程扎实推进，市文化馆被评为“国家一级馆”。《李之仪研究论文集》出版发行，黄梅戏音乐电视剧《诗仙李白》完成拍摄试播。数字电视整体转换顺利推进。“迎奥运”全民健身系列活动广泛开展，社区和农民体育健身工程加快建设。市档案馆成为全省首家“国家二级综合档案馆”。《马鞍山市志》续志编纂工作全面完成。文明创建工作成绩显著?础上，一举跻身第二批全国文明城市候选城市行列。

    教育、卫生工作取得新成绩，各项社会事业全面进步。在全省率先对城市义务教育阶段学生免费提供教科书，减免民办学校义务教育阶段学生杂费，放宽外来务工人员子女就学政策。花园中学主体工程基本完成，特教学校建成投入使用。职业技术学院更名和迁址新建工程如期完成，中职教育集团顺利组建。河海大学文天学院一期工程建成并完成首届招生，安工大、马鞍山师专办学质量和水平进一步提高。市人民医院、中心医院被评为“三甲”医院，人民医院门急诊大楼开工建设，雨山区“国家社区卫生服务适宜技术”试点进展顺利。全面实施免费婚前医学检查，209名贫困白内障患者接受免费手术成功复明。血吸虫病防治工作取得阶段性成果，县区分别达到疫情和传播控制标准。农村计划生育奖励扶助制度有效落实，企业领证退休职工一次性奖励补助全部兑现，人口出生率控制在10.2‰以内。全民科学素质行动计划启动实施。第二次经济普查全面展开。人防工作走在全省前列，国防动员、民兵预备役部队建设积极推进，“全国双拥模范城”正式授牌。国家一级气象站主体工程封顶，防震减灾扎实开展。民族宗教工作卓有成效。对台经贸、文化交流不断增多。妇女、儿童、老龄人和残疾人事业取得新进展。

    （八）尽最大努力保障公共安全，较好维护了社会和谐稳定。过去一年，面对一系列重大突发事件，全市上下众志成城，共克时艰，打赢了一场场硬仗。在抗击年初特大雨雪冰冻灾害过程中，我们周密部署，迅速行动，在最短时间内实现了保畅通、保安全、保生活、保供应、保生产的目标，把灾害造成的损失降到了最低程度。面对突如其来的手足口病和流行性腮腺炎疫情，我们立足早发现、早送诊、早治疗，迅速开展疫苗接种，有效预防和控制了疫情蔓延。汶川特大地震发生后，我们组织企业紧急生产救灾物资，及时派出医疗防疫、过渡安置房建设专业人员，千里驰援灾区，并对灾区来马学生进行妥善安置。全市人民自发捐赠，无私奉献，涌现出许多心系灾区、情系巴蜀的感人事迹。三鹿奶粉重大安全事故发生后，我们及时派出工作组分别进驻乳制品生产企业、商场和医院，实行企业生产全程监管和产品批批检验，督促商场全部下架封存不合格产品，并对已售问题奶粉及时退款，对全市3万多名5周岁以下婴幼儿免费开展筛查、诊断和治疗，妥善化解了问题奶粉可能引发的突出矛盾。

    坚持高标准、严要求，全面落实奥运安保重大政治任务。认真开展大接访和赴京接访劝返工作，加大重点信访案件专项整治和督查督办力度，顺利实现中央和省委、省政府提出的工作目标，成为全省信访维稳形势较好城市之一。派出2批13人次赴京参与奥运会、残奥会食品安全保障工作，出色完成各项任务。强化社会治安综合治理，校园周边等突出治安问题整治、矛盾纠纷排查调处成效明显，以老旧小区安全防范设施为重点的治安防控体系建设稳步推进，人民群众安全感高于全省平均水平，被评为“全省社会治安综合治理先进城市”。人民调解百日会战、社区矫正和安置帮教工作取得积极成果，“五五”普法工作稳步实施。全面整顿矿产资源开发秩序，扎实开展安全生产隐患治理、百日督查和专项检查，强化安全生产基础工作，全市安全生产形势保持平稳。组织编写并免费发放市民应急避险防灾、居民安全防范手册，积极开展应急预案演练，公民预防和应对突发事件能力得到提高。

    一年来，市政府坚持党的领导，坚决贯彻落实市委作出的各项决策部署。认真执行市人大及其常委会决议、决定，定期向市人大常委会报告工作和向市政协通报情况，主动接受市人大的法律监督、工作监督和市政协的民主监督。政府系统承办的11件人大代表议案、148件建议批评意见、328件政协提案全部办复。按照市委的统一部署和要求，政府系统深入学习贯彻科学发展观，扎实开展新一轮解放思想大讨论活动，市政府负责同志牵头整改的12个重难点问题取得明显成效。修订完善全面小康社会评价指标体系，明确年度目标任务，逐项分解落实到位，全面小康进程加速推进。加大重点工作督查力度，开展社会公众评议机关活动，目标管理考核进一步强化。加快法治政府指标体系建设，规范行政处罚自由裁量权，依法行政工作得到加强。修订完善市政府工作规则，建成政府信息公开平台，电子政务建设步伐加快，政风行风热线联系群众的桥梁作用充分发挥。强力实施行政审批“两集中、三到位”工作，基本完成职能归并、窗口授权、机构调整和人员选派，54个部门388项审批事项进驻到位。完善公务员考核办法，机关效能建设岗位禁令颁布实施。强化税源管理和纳税服务，财政增收节支工作得到加强。实行政府投资建设项目审计主动申报制，民生工程和经济责任审计范围进一步拓展。首次向政府投资重点项目和民生工程派驻监察组，反腐倡廉制度建设推进年活动取得积极成果。

    在肯定成绩的同时，我们也清醒地看到，当前宏观经济环境的急剧变化，更加暴露出我市经济社会发展中一些突出的矛盾和问题，特别是经济结构不优、产业层次不高、自主创新能力不强，部分行业和企业应对风险能力较弱等问题尤为明显。社会事业领域改革有待深入，社会管理水平尚需提高。城乡居民实现充分就业、持续增收难度不断加大，推进城乡一体化发展的任务还十分艰巨。政府自身建设还存在一些薄弱环节，机关作风和效能建设需要进一步加强，消极腐败现象还不同程度地存在。对此，我们将高度重视，采取有效措施，切实加以解决。

    各位代表，过去一年，我们发展的道路很不平坦，取得的成绩确实来之不易。这是省委、省政府和市委正确领导的结果，是全市上下同心协力、顽强拼搏的结果。在此，我代表市人民政府，向辛勤工作在各条战线上的广大干部群众，向给予政府工作大力支持的市人大代表、市政协委员，各民主党派、工商联和无党派人士，各人民团体，致以崇高的敬意！向参与、支持马鞍山现代化建设的中央和省驻马单位、解放军和武警官兵、海内外朋友，表示衷心的感谢！

    二、坚定信心、迎难而上、群策群力做好2009年工作

    2009年是新中国成立六十周年，也是我市推进全面小康建设的关键一年。去年下半年以来，受国际金融危机快速蔓延、国内经济增长明显减速的不利影响，我市企业生产经营困难增多，企业效益大幅下滑，经济下行压力明显加大。当前，这场历史罕见、冲击力极强、涉及范围很广的国际金融危机不仅本身尚未见底，而且对实体经济的影响正进一步加深。外需萎缩和内需不振同时出现，支撑全市经济发展的工业、投资、出口等主要因素将发生新的变化，势必给钢铁、汽车、机械、化工等支柱产业造成更大冲击，我市发展面临的形势异常复杂严峻。如何积极有效地应对来自国际国内的不利影响，千方百计保持来之不易的发展势头，将是今年我们面临的最大挑战、最大考验，也是政府工作的首要任务、头等大事。

    我们更要看到，这一年也是蕴含重大发展机遇的一年。中央把保持经济平稳较快发展作为今年经济工作的首要任务，实施积极的财政政策和适度宽松的货币政策，采取一系列扩大内需促进增长的有力措施，为我市扩大投资、调整结构、增强后劲、改善民生带来了前所未有的机遇。同时，金融危机形成的倒逼机制，既迫使我市更多企业加快转型升级，也为一些有实力的企业实施低成本扩张提供了机会。随着沿海地区经济加速转型和产业转移步伐加快，特别是泛长三角区域合作机制的建立，有利于我们发挥综合比较优势，进一步拓展发展空间。经过新世纪以来快速发展，我市积累了较为雄厚的物质基础，造就了一批具有较强核心竞争力的骨干企业，政府调控能力和经济抗风险能力显著增强。尤其是广大干部对科学发展观的认识不断深化，结合实际创造性开展工作的经验更加丰富。只要我们既充分估计困难、周密准备措施，又善于捕捉机遇、抓住有利条件，坚定信心，知难而进，迎难而上，就完全能够战胜暂时的困难，进而赢得更长时期、更高水平、更好质量的发展。

    做好今年各项工作，我们要全面贯彻党的十七大、十七届三中全会和中央、全省经济工作会议精神，以邓小平理论和“三个代表”重要思想为指导，深入贯彻落实科学发展观，突出扩大有效投入促进经济发展，突出推进自主创新转变发展方式，突出深化改革开放增强发展活力，突出城乡统筹加快一体化步伐，突出改善民生加强社会建设，努力实现经济社会又好又快发展，奋力向“又好又快新跨越、率先全面达小康”目标迈进。

    综合考虑各种因素，今年经济社会发展的主要预期目标为：生产总值增长10%以上；财政收入与生产总值同步增长；全社会固定资产投资增长20%以上；社会消费品零售总额增长12%以上；城市居民人均可支配收入、农民人均纯收入均增长8%以上；城镇登记失业率控制在4%以内；万元生产总值综合能耗下降4.3%；主要污染物排放量、人口自然增长率均控制在省下达指标以内。实现这一目标，对于进一步缩小与长三角的差距、实现在省内率先发展，对于增强信心、稳定预期、扩大城乡就业和保持社会稳定，十分必要。但在国际、国内经济增长明显减速的情况下，完成这一目标并不容易，需要我们付出艰苦的努力。在实际工作中，我们要立足于能快则快，确立更高的奋斗目标，排除万难争取实现。

    实现全年经济社会发展目标，我们必须把保增长、抓项目、帮企业、扩内需、调结构、增活力、促民生、构和谐贯穿始终，下大气力重点抓好八个方面工作：

    （一）千方百计扩大投资和消费，为经济平稳较快发展提供强力支撑。牢牢把握中央宏观政策导向，紧密结合我市实际，最大程度地发挥扩大内需政策效应，强化经济增长的支撑力。

    加大项目谋划和争取力度。牢固树立抓项目就是抓投资、抓投资就是保增长的意识，把提升项目工作水平作为经济工作的第一位任务，切实抓紧抓实抓出成效。对已上报国家发改委的项目，全方位加强衔接，锲而不舍地盯紧盯实，同时做好各项准备工作，确保项目一旦获批立即开工，为后续资金争取创造条件。对尚未列入国家发改委投资计划的项目，积极主动跑省跑部，及时把握项目动态，全力争取业务指导和申报审批支持。深入谋划大唐当涂电厂二期、华菱新一代环保大功率发动机、山鹰80万吨板纸和文化用纸、方圆风电和重装回转支承等大项目，抓紧完善规划、土地、资金、环评等前期工作。同时，着眼于打基础、管长远、增后劲，加快储备一批城市基础设施、社会事业、服务业、工业交通能源等项目，完善和充实项目储备库。

    集中力量推进重点项目建设。健全以项目为中心，上下联动、责任明确、高效运作的推进机制，确保能开工的及早开工、能加快的尽量加快、能竣工的早日竣工。开工建设马钢“十一五”后期结构调整项目，推进长江钢铁等企业460万吨钢铁产能置换项目建设，促进钢铁产业加速向千亿级迈进。开工建设马鞍山发电厂两台60万千瓦机组项目，启动华菱3万辆重卡扩建工程，推动科达机电和雨润、达利食品二期项目建设，加快立白化工、中橡炭黑扩能项目进度，促进电力、汽车、食品、化工等产业加速向百亿级冲刺。加快泰尔重工高性能联轴器项目和天成纺织搬迁改造步伐，支持矿院微珠高性能新材料项目建设，扩大机械制造、纺织服装、新材料产业规模。支持县区加快实施凯迪生物质能发电、双力起重设备、格瑞德工程机械、锐生工贸传送带等一批项目，不断增强县区工业发展后劲。

    大力推动服务业优化升级。充分发挥区位、交通等综合优势，加快长运物流港、华东物流园建设，促进物流业快速发展。建成大华国际广场二期和沃尔玛大型超市，打造新都市场商业圈，积极培育消费热点。理顺采石风景区旅游管理体制，全面整合旅游资源，统一实施综合开发，带动全市旅游业加快发展。进一步完善地方金融体系，在市农村商业银行、浦发银行马鞍山支行尽快挂牌运营的基础上，争取新设村镇银行和其他金融机构，组建2家规模较大的小额贷款公司，促进保险、证券、期货、典当行业加快发展。认真落实稳定和发展房地产业相关政策措施，合理引导住房消费和房地产开发建设，保持房地产市场健康发展。加大保障性住房建设力度，开工新建廉租房、城市拆迁安置房各1000套，实施危旧房改造20万平方米。积极开展“家电下乡”活动，增加农村市场适销对路商品供应，挖掘农村消费潜力。

    （二）全力以赴为企业排忧解难，进一步强化工业经济主动力作用。企业是经济增长的主要源泉，保增长首先要保企业。要扎实开展“企业帮扶年”活动，支持企业在战胜困难中发展壮大、做优做强。

    加强工业经济运行调节。强化工业运行监测预警，有针对性地帮助重点行业、骨干企业解决面临的困难和问题。对目前市场需求旺盛的优势工业产品，加大资金支持和政策扶持力度，促进企业开足马力扩大生产。鼓励各类企业利用市场竞争的倒逼机制，苦练内功，深挖潜力，在技术、产品和管理创新上迈出更大步伐，不断增强企业核心竞争力。充分调动广大职工的主观能动性，与企业同呼吸共命运，凝心聚力共渡难关。积极创建全国优质机床及刃模具生产示范区，进一步提升冶金装备制造、磁性材料等产业集群发展层次，增强产业集聚效应。力争全年新增规模以上工业企业超100户、销售收入超亿元企业10户，规模以上工业增加值增长15%以上。扶持建筑业企业做大做强，促进建筑业持续健康发展。

    着力缓解企业发展资金难题。充分发挥银行信贷主渠道作用，支持、引导银行机构加强和改进信贷服务，更多地推出适合中小企业特点的金融产品，满足企业合理资金需求。创新银政、银企合作方式，加大优质企业推介力度，鼓励通过银团贷款等方式支持大企业、大项目。支持金融创新，探索开展股权、知识产权、应收账款和仓单质押融资。按照党的十七届三中全会关于“扩大农村有效担保物范围”的要求，在农村探索开展经合法登记的农民住房抵押贷款。放大中小企业发展专项资金效应，在企业新上技术改造、短期融资贷款等方面实行奖补和贴息支持。进一步扩大中小企业贷款风险补偿范围和担保公司资本金规模，拉动金融机构对中小企业的信贷投放。出台上市公司培育办法，推动泰尔重工、鼎泰稀土加快上市融资，支持华菱西厨、奥盛钢缆、黄池食品等企业做好上市准备，新增上市公司2家以上。

    深入推进全民创业。认真落实发展非公有制经济的政策措施，发挥政府扩大投资对民间投资的拉动作用，充分调动各类创业主体的积极性、主动性。继续清理和减免行政事业性收费，实行创业初期“零收费”。加快各类创业载体建设，完善创业园区服务功能，新建创业基地15万平方米。逐级建立小企业及个体工商户发展情况信息测报点制度，强化帮扶措施，努力提高创业成功率。开工建设新的中小企业服务中心，完善社会化服务体系，重点在资金、用地、技术、人才等方面加大扶持力度，形成全社会鼓励、支持、尊重创业的浓厚氛围。

    （三）以城乡一体化综合配套改革为抓手，全面推动农村改革发展。坚持城乡统筹，把建设新农村作为战略任务，把加快形成城乡一体化新格局作为根本要求，以体制机制创新为动力，努力实现农村改革发展新突破。

    全面实施城乡一体化综合配套改革总体方案和专项方案。花山区要加快试点步伐，基本完成张庄中心村建设任务。其他县区要因地制宜，力争在促进农业用地向规模经营集中、农村工业向园区集中、农民就业向二三产业集中、农村人口向城镇集中上取得积极进展。建立土地流转服务中心，开展不同类型农村土地承包经营权流转试点，促进农村土地适度规模经营。全面完成集体土地上房屋权属登记发证工作。深入推进集体林权制度配套改革，促进集体林权流转。探索设立农村资金互助合作社和农业贷款担保公司，继续做好政策性农业保险试点工作，完善农村金融服务功能。进一步加强农村基层组织建设，深化村务公开，完善村级财务管理，不断扩大基层民主。

    大力发展现代农业。制定扶持政策措施，实施农业产业化提升行动规划，促进高效规模特色农业加快发展。以培育龙头企业和发展农民专业合作组织为重点，加快优势农产品基地建设，完善龙头企业与农户的利益联结机制，提高农业生产规模化、标准化和组织化水平。力争全年营销收入超亿元企业达8家，建成农业标准化示范基地10个，新增无公害农产品、绿色食品和省级以上名牌农产品6个。以农产品精深加工、规模化养殖和休闲观光项目为突破口，进一步扩大农业对外开放，全年引进农业“三资”4亿元以上。抓好当涂县国家级万亩水稻高产创建示范区和油菜高产攻关示范项目建设，加快农业科技推广应用，确保粮食稳定增产。实施新农村科学储粮示范工程，进一步增加市级粮食储备规模。推进“新网工程”建设，鼓励发展各类农业生产经营服务组织，构建新型为农服务体系。建立农产品质量安全可追溯制度，加强重大动植物疫病防控，确保农产品质量安全。

    多渠道增加对农村公共服务的投入。继续推进乡镇综合文化站和村文化室、农家书屋建设，优化调整农村学校布局。加大农村卫生人才培养力度，强力推进乡镇卫生院、村卫生室标准化建设。按规划要求推动“村村通”水泥路向村民组延伸，争取新增通村公路80公里，行政村班车通车率达95%。加强农田水利设施建设，继续实施病险水库除险加固、涵闸除险改造工程，新建慈湖河泵站。建立城乡统一的就业、失业登记与管理制度，落实农民工培训补贴、养老保险关系转移接续政策，有组织地促进农民就近就地就业。积极引导和扶持农民创业，以创业带动就业稳定增长，努力缓解农村就业压力。

    （四）坚定不移地推进改革开放，为科学发展增创体制机制新优势。实践证明，改革开放是加快发展的强大动力和必由之路。越是困难较多的时候，越要毫不动摇地坚持深化改革和扩大开放，不断增强发展的动力和活力。

    着力深化重点领域和关键环节改革。按照统一部署，推进新一轮政府机构改革，进一步转变政府职能。坚持事权、财权相统一，实施新的市区财政管理体制，完善收入征管体系，推动三区进一步加快发展步伐。积极探索和完善县域经济发展新体制。全面推行事业单位分类改革和人员聘用制度，有序实施岗位设置管理工作，不断深化事业单位分配制度改革。推进公益性文化事业单位管理体制、运行机制改革，加快经营性文化单位转企改制步伐，基本完成文化体制改革主要任务。加强行政事业单位资产管理，组建行政事业资产管理公司。稳妥做好工商系统市场办管脱钩后的相关工作，促进集贸市场健康发展。扎实推进水、电等资源性产品价格改革，开展污水、垃圾处理和排污收费改革。进一步完善油运价格联动机制，确保成品油税费改革平稳实施。

    切实增强招商引资的针对性和实效性。深入研究把握国内外产业重组与转移新特点，采取更加灵活的政策措施，加大先进制造业、高新技术产业、服务外包业招商引资力度，努力引进世界500强、中央直属企业和国内上市公司。着力构建市场化、专业化、网络化招商平台，推动产业集群招商、产业链招商，完善招商绩效考核机制，保持实际利用内外资持续增长。继续支持三个省级开发区加快发展，进一步增强承载功能，提升产业配套能力，改善综合商务环境，推进“二次创业”。积极争取市经济技术开发区升格为国家级开发区、花山商贸旅游园升格为省级开发区、博望工业集中区升格为国家级特色产业园，推动各类开发园区发挥优势错位发展。

    积极扩大对外经贸和区域合作。综合运用出口退税、外贸发展基金、财政贴息等政策措施，加大新兴市场开拓力度，打造出口产业基地，力争全年出口额增长15%以上。支持引导马钢、十七冶、中冶华天等优势企业以总承包方式对外承接大工程，扩大对外劳务合作，开展境外资源开发和企业并购，不断提升参与国际竞争的能力。进一步完善口岸功能，实现口岸对外籍轮开放，扩大进出口货物属地报关、就地直运量。紧抓泛长三角区域分工与合作的机遇，努力在皖江城市带承接产业转移示范区中发挥先导作用，加快形成对内对外开放相互促进、国际国内市场全面拓展的新格局。

    （五）着力抓好自主创新和节能减排，促进经济发展方式加快转变。自主创新和节能减排是转变发展方式的重要途径。要利用金融危机形成的倒逼机制和经济调整产生的洗牌效应，在自主创新和节能减排上迈出更大步伐。

    加强自主创新能力建设。比照合芜蚌自主创新综合配套改革试验区政策规定，出台促进自主创新具体措施，引导全社会加大科技投入。紧紧围绕我市支柱产业和骨干企业升级发展，实施新能源应用、新材料等9个重大专项计划，不断提高科技对产业升级的支撑力。发挥企业在自主创新中的主体作用，推动多种形式的产学研合作，促进各类创新要素向企业集聚，争取更多企业进入创新型企业行列。加快花山软件园、雨山佳达创意软件园、茂文科技园建设，着力培育环保、生物工程等新兴产业和高新技术产业，形成一批具有爆发性潜力的经济增长点，带动产业结构向更高水平迈进。完善市科创中心服务功能，加快国家钢铁及制品质量监督检验中心建设步伐，建成国家矿产资源高效循环利用工程技术中心，充分发挥国家矿山固体废物处理与处置工程技术中心平台作用。积极争创国家知识产权试点市。全年力争研发经费占生产总值比重达到1.5%，新增高新技术企业5家以上，科技进步对经济增长的贡献率提高2个百分点。

    切实抓好能源资源节约和生态环境保护。支持马钢新区高炉鼓风脱湿系统节能优化改造和山鹰造纸污泥发电等项目建设，推动重点企业新建、扩能污水处理工程尽快实施。加快东部污水处理厂、慈湖污水处理厂、开发区南区污水处理厂、当涂污水处理厂建设步伐，进一步提高城市污水处理率。认真落实重点行业和重点企业节能减排任务，强化新建项目环境容量管理，确保完成节能减排目标。坚持节约集约用地，严格执行耕地占补平衡和基本农田保护制度。推进建设用地置换和城乡建设用地增减挂钩工作，有效拓展建设用地空间。

    （六）坚持扩张外延与丰富内涵并重，努力提升城市功能和品位。围绕建设“双百”现代化城市，拉开城市发展大构架，不断改善城乡人居环境，实现承载能力、城市品位双提升。

    全面构建综合交通运输体系。加快推进长江公路大桥建设，同步实施桥塔、引桥及南北接线、江心洲洲头保护工程。推进宁宜城际铁路及配套工程建设，加快芜申运河航道整治工程步伐。实施东环路高速化改造，扎实推进马濮路改造升级。加强港口码头建设，全面提升港口吞吐能力。通过一批重大交通基础设施项目建设，进一步畅通连接长三角、皖江城市带的快速通道。同时，改扩建一批县乡道路，不断改善市域内交通整体状况。

    加强城市基础设施建设。推进印山路和湖南路东延、霍里山大道北接，新建九华东路，进一步扩展城市东部路网。延伸湖东南路，打通南进当涂县城的又一快速通道。加快慈湖河路北延步伐，完善城市北部地区路网。开工建设沿江大道一期工程，逐步改善城市西部路况。加强城市出入口综合整治，提升城市窗口形象。建设湖北路滨湖休闲游园，增加公共绿地面积，提升城市园林绿化水平。完善博望、石桥等中心镇功能，全年完成小城镇建设投资16亿元。加强城市水环境治理，重点整治西南排水系。全面开工建设12个电网项目，不断提高城乡电网供电能力。加强通信基础设施建设，提升通信服务能力。鼓励和支持公交优先发展，规范和加强出租车行业管理，进一步方便市民出行。

    持之以恒抓好城市长效管理。巩固和发展创建全国文明城市工作成果，不断提高全国绿化模范城市水平，扎实做好国家环保模范城、国家卫生城市、国家园林城市迎检复核工作。加快编制城乡空间布局规划和专业规划，探索建立市、县区、乡镇三级规划管理体系，加强城乡建设管理。强化市容环境卫生考核和违法建设责任追究，进一步提升城市管理水平。全面实施慈湖河流域生态环境综合整治，基本完成污染治理和河道清淤工程，启动水土保持、绿化复垦工程建设，加快恢复向山地区、慈湖河两岸的生态面貌。全力推进金家庄部分地区居民搬迁工作，年内搬迁江边地区居民1500户。继续推进老旧小区改造，进一步规范小区物业管理。继续做好“油改气”相关配套工作，逐步建立全市机动车排气污染防治与监控体系。开工建设危险废物集中处置中心、有机物处理中心和环境监控中心，促进环卫基础设施逐步完善。
   
    （七）健全民生工程长效机制，在更高水平上保障和改善民生。完善民生工程长效机制，是解决人民群众最关心、最直接、最现实利益问题的根本保障。我们要在认真总结经验的基础上，进一步加大投入、健全机制，保障民生工程在相对稳定的制度轨道上运行。

    全方位促进就业增长。根据就业形势新变化，实施更加积极的就业政策，调动社会各方面力量，支持有利于扩大就业的劳动密集型产业、中小企业和服务业发展，千方百计增加就业岗位。鼓励企业采取在岗培训、轮班工作、协商减薪等办法，努力做到不裁员或少裁员。强化城乡困难群体就业援助，尽可能多地开辟公益性岗位。扎实推进“消除零就业家庭行动计划”，统筹做好高校毕业生、??市场和人才市场，建成当涂县人力资源市场和乡镇分市场，健全完善覆盖城乡的公共就业服务体系。积极开展就业再就业培训，推动技能人才队伍不断壮大。全年新增城镇就业2.4万人，新增农村劳动力转移就业1.5万人，完成职业技能培训1万人。深入实施劳动合同制度和劳动用工登记制度，努力发展和谐劳动关系。

    进一步提高社会保障水平。继续完善城镇职工基本养老、失业、医疗、工伤、生育保险，逐步扩大医疗保险单病种结算范围，将个体工商户纳入工伤保险保障范畴。深入推进新型农村养老保险参保扩面，扎实做好城镇居民养老保险工作，加快实施事业单位养老保险制度，完善城乡居民基本医疗保险制度，实现养老保障、医疗保险城乡居民全覆盖。积极推进社会救助体系建设。加快城乡低保统筹步伐，制定低收入阶层和特殊弱势群体救助办法，调整城乡医疗救助封顶标准。建立农村五保供养标准自然增长机制，推进农村敬老院改扩建工程和城市老年公寓建设。大力发展慈善事业。

    着力提升“平安马鞍山”建设水平。深入推进社会治安综合治理，继续加强治安防控体系建设，全面落实流动人口服务和管理措施，夯实基层基础工作。强力推进公安信息化建设应用，探索实践警务机制改革，使刑事案件发案数稳中有降、打击处理数稳中有升。继续深入开展法制宣传教育，推进司法所规范化、信息化建设，扎实做好法律援助、社区矫正和刑释解教人员安置帮教工作，促进人民调解创新发展。进一步健全维护社会稳定工作机制，建立社会稳定风险评估和信访社情分析、评估、救助制度，努力化解信访矛盾，切实维护全市治安平稳和社会稳定。坚持安全发展，层层落实政府监管责任和企业主体责任，强化隐患排查治理，防范和遏制重特大安全事故发生。切实抓好食品药品质量安全，加强日常监管和专项整治，健全保障质量安全的长效机制。加快完善应急管理体系和工作机制，提高保障公共安全和处置突发事件的能力。

    （八）大力促进公共服务均等化，不断提高社会事业整体发展水平。加快发展社会事业是促进科学发展的重要内容。要进一步优化公共资源配置，推动社会事业全面进步，形成经济社会协调发展新格局。

    优先发展教育事业。改革义务教育管理体制，实行市直属小学和初中下放区级管理。大力普及高中阶段教育，实现全市高中阶段教育毛入学率达84%以上。开工建设南部新城高级中学，确保二十中迁址新建主体工程封顶。全面启动职业教育现代化工程，组建高职集团，开工建设职教园区，加快现代服务、汽车运用与维修实训基地建设步伐，建成数控实训基地。支持安工大东校区二期和河海大学文天学院二期工程建设，扎实做好新引进高校工作。认真实施义务教育等学校绩效工资制度改革，实现高中阶段学校资助体系全覆盖。

    推动文化繁荣发展。扎实推进社会主义核心价值体系建设，增强社会主义意识形态的吸引力和凝聚力。全面开放博物馆、新图书馆，基本建成大剧院，进一步完善文化基础设施。实施文化精品工程，办好’2009马鞍山中国李白诗歌节，举办首届骏马奖游戏动漫全国大赛。加强新闻和网络文化管理，繁荣发展文学艺术和哲学社会科学。加强李白文化深层次研究，推动文化与旅游等相关产业融合，赋予城市建设更多的文化内涵。申报太白楼为全国重点文物保护单位，做好全国第三次文物普查工作，保护和利用好文化遗产。

    增强公共卫生服务保障能力。深化市立医疗集团内部管理体制改革，进一步完善运行机制，改善医疗服务质量，提高医疗技术水平。加快城乡卫生服务体系建设步伐，增强基层医疗服务能力。鼓励社会资本参与投资医疗卫生服务领域，积极引导民营医疗机构发展，形成多元化办医格局。加强突发公共卫生事件医疗救治体系、卫生监督执法体系、疾病预防控制体系建设，增强重大传染病、地方病及人畜共患病防控能力，推动爱国卫生运动深入开展。积极创造条件，争取进入全国医疗卫生体制改革试点市行列。

    统筹发展各项社会事业。开工建设集科技馆、青少年宫和妇女儿童活动中心为一体的综合馆，加快体育中心前期工作步伐。启动新广电中心建设，完成数字电视整体转换工程。提高竞技体育水平，推动群众体育广泛开展。全面落实计划生育奖励优惠政策，进一步稳定低生育水平。深入实施全民科学素质行动计划。积极探索社区管理模式，开展特色社区创建工作，稳妥推进农村社区建设试点，着力构建和谐社区。深入开展城乡社会志愿服务活动，充分发挥各类团体在社会建设中的重要作用。依法管理民族宗教事务，扎实做好新形势下民族宗教工作。重视发展老龄人、残疾人事业，切实保障妇女和未成年人权益。推进外事、侨务和对台工作健康发展，加强气象、防震减灾和档案、地方志等工作。支持驻马部队建设，加强国防动员和人防工作，加快民兵预备役综合训练基地建设步伐，深入开展拥军优属和军民共建活动，巩固发展军政军民团结的良好局面。

    三、励精图治、务实创新、善谋善为驾驭复杂局面

    当前面临的复杂局势和艰巨任务，对政府自身建设提出了更高要求。我们一定要深入学习实践科学发展观，树立强烈的责任意识和进取意识，以更加昂扬的斗志、更加扎实的作风、更加有为的工作，从容应对各种挑战，勇于战胜各种困难，推动经济社会又好又快发展。

    坚持用科学发展观武装头脑、指导实践。全市政府系统要按照市委的统一部署，把开展深入学习实践科学发展观活动作为一项重大政治任务，认真领会科学发展观的深刻内涵、精神实质和根本要求，着力转变影响和制约科学发展的思想观念和体制机制，切实提高驾驭市场经济和应对复杂局面的能力。尤其要把学习实践活动与保持经济平稳较快发展紧密结合起来，坚持学以致用，着力把握发展规律，创新发展理念，转变发展方式，破解发展难题，实现发展目标，做到既注重即期增长，又注重发展后劲；既注重增长速度，又注重发展质量；既注重经济建设，又注重社会和谐；既注重谋求发展，又注重改善民生。

    始终保持迎难而上、奋发有为的精神状态。危机和困难面前考验的是信心，是能力，更是意志。我们要时刻保持逆水行舟的紧迫感、知难而进的精气神，只争朝夕，埋头苦干，以饱满的激情投入到各项工作中。要坚定战胜困难的信心和决心，不气馁、不退缩。要坚持迎难而上，善于出招，敢于“亮剑”，攻下难点，闯过难关，夺取胜利。尤其是要进一步解放思想，只要是对科学发展有利、对群众利益有利的事情，看准了就大胆地试、大胆地闯、大胆地干，以更大的气魄加快发展，以更实的举措推动发展，努力保持率先发展、科学发展、和谐发展的良好势头。

    进一步健全科学决策、民主决策机制。越是在困难和复杂的情况下，越是要听取民声，顺应民意，做到科学民主决策。市政府将更加自觉地接受市人大及其常委会的法律监督、工作监督和市政协的民主监督，认真听取各民主党派、工商联、无党派人士及各人民团体的意见，广泛接受社会公众和新闻舆论监督。不断完善行政决策专家论证、社会听证和合法性审查制度，建立行政决策评估机制，促进各项决策民主化、科学化。政府系统各级干部要腾出更多的精力深入实际、深入基层、深入群众，开展调查研究，做到问政于民、问需于民、问计于民，使提出的发展思路、工作部署、政策措施更加切合实际，促进各项工作落到实处。

    着力营造务实高效、依法行政的发展环境。保持经济平稳较快发展，迫切要求进一步规范行政行为，优化政务环境。全市政府系统要大兴真抓实干之风，力戒形式主义、官僚主义，不搞花架子，不做表面文章。巩固“两集中、三到位”工作成果，进一步调整和减少行政审批事项，优化审批流程，提高办事效率。大力推进政府信息公开，加强电子政务建设，以公开促规范，以公开促自律，以公开促监督。强化政务督查，健全以绩效考核为导向的目标管理体系，认真落实效能建设各项制度，切实提高政策执行力和工作落实力。全面推进依法行政，细化量化行政裁量权，严格行政执法责任制和过错责任追究制，促进行政执法监督制约机制进一步完善。

    更加注重树立勤俭办事、廉洁从政的政府形象。俭以养德，廉以立身。政府系统要带头过紧日子，严格压缩会议、接待、出国经费和各项行政开支，积极创建节约??健全完善教育、制度、监督并重的惩治和预防腐败体系，认真落实党风廉政建设责任制和领导干部廉洁自律预警机制，巩固扩大反腐倡廉制度建设推进年活动成果，从源头上防治腐败。严格执行政府投资项目招投标、专项资金使用管理和政府采购等制度，切实加强对中央投资项目和资金的管理与监督检查。强化行政监察、审计监督，进一步规范公共资源管理权力运行。政府系统工作人员要始终牢记“两个务必”，当好清廉从政的表率，以实际行动取信于民。

    各位代表，困难和挑战考验着我们，责任和使命激励着我们。让我们高举中国特色社会主义伟大旗帜，深入贯彻落实科学发展观，在省委、省政府和市委的正确领导下，紧紧依靠全市人民，齐心合力，顽强拼搏，攻坚克难，努力开创各项事业发展新局面，奋力夺取全面小康建设新胜利，以优异成绩迎接建国六十周年！
